# Posterior-only debridement, internal fixation, and interbody fusion using titanium mesh in the surgical treatment of thoracolumbar tuberculosis with spinal epidural abscess: a minimum 5-year follow-up

**DOI:** 10.1186/s12891-021-04797-2

**Published:** 2021-11-01

**Authors:** Qile Gao, Chaofei Han, Manini Daudi Romani, Chaofeng Guo, Mingxing Tang, Yuxiang Wang, Ang Deng, Shaohua Liu, Hongqi Zhang

**Affiliations:** 1 Department of Spine Surgery, Xiangya Hospital, Central South University, No. 87, Xiangya Road, Changsha, 410008 People’s Republic of China; 2 National Clinical Research Center for Geriatric Disorders, Xiangya Hospital, Central South University, Changsha, China; 3 Department of Burn and Plastic Surgery, The third Xiangya Hospital, Central South University, Changsha, China

**Keywords:** Spine tuberculosis, Thoracolumbar, Posterior approach, Titanium mesh

## Abstract

**Objective:**

To investigate the clinical efficacy and feasibility of posterior-only debridement, internal fixation, and interbody fusion using titanium mesh in the surgical treatment of thoracolumbar tuberculosis (TB) with spinal epidural abscess.

**Methods:**

From January 2008 to January 2014, a total of 45 patients (27 male and 18 female) were reviewed. The patients were diagnosed with thoracolumbar TB with spinal epidural abscess. The patients underwent posterior-only debridement, internal fixation, and interbody fusion using titanium mesh. Hence, we assessed the intraoperative and postoperative complications, disease recurrences, kyphosis deformity correction, and neurological improvement following the American Spinal Injury Association (ASIA). We used SPSS 22.0 for the statistical analyses. An independent Student’s t-test was used for the analysis of preoperative and postoperative continuous variables. The value of P (*P* < 0.05) was considered statistically significant.

**Results:**

The mean age of patients was 37.76 ± 10.94 years (17–59 years). The mean follow-up time was 82.76 ± 12.56 months (60–128 months). The mean kyphosis Cobb angle preoperative was 29.36 ± 13.29° (5–55°) and postoperative was 3.58 ± 5.44° (− 6–13°), given the value of P (*P* < 0.001). According to the neurological score by the ASIA scale, there were 3 cases of grade B, 11 cases of grade C, 16 cases of grade D, and 15 cases of grade E preoperatively. The neurological score improved by 1 ~ 2 grades. All patients achieved pain relief and the VAS score significantly reduced at the last follow-up (P<0.05). While 1 patient had cerebrospinal fluid leakage, 1 had a neurological complication, 1 had delayed surgical wound healing, and 1 had a disease recurrence. No pseudoarthrosis or implant failure occurred in our patients. All patients achieved solid bone graft fusion.

**Conclusion:**

For thoracolumbar TB patients with spinal epidural abscess, posterior-only debridement, internal fixation, and interbody fusion using titanium mesh are safe and effective surgical treatments.

**Supplementary Information:**

The online version contains supplementary material available at 10.1186/s12891-021-04797-2.

## Introduction

Spinal tuberculosis (TB) also called Pott’s disease, is the most common and severe form of osteoarticular TB. Usually, if TB remains uncontrolled after conservative treatment, then surgery is considered as an effective treatment for patients affected by bone destruction, angular deformity, spinal instability, paraspinal or spine canal abscess, or nerve impairment. The surgical strategy for the treatment of Pott’s disease is to meticulously debride the diseased lesion, provide standard anti-tuberculosis chemotherapy, alleviate the symptoms of nerve compression, correct kyphosis deformity, and restore spinal stability.

The thoracolumbar spine is a transition zone between the relatively stable thoracic vertebrae and the highly movable lumbar vertebrae. Thoracolumbar spine TB, in combination with the spinal canal abscess, can easily induce nerve damages caused by spinal cord compression. For thoracolumbar spine TB patients, active surgical treatment is recommended. Anterior debridement, bone grafting, and anterior- or posterior- internal fixation operations have been considered as the standard methods for the treatment of spinal TB; however, these methods always lead to substantial surgical trauma [1, 2].

For patients with kyphosis and intraspinal space-occupying lesions, posterior-only surgery can simplify the operation and give a clinical effect similar to anterior or anteroposterior surgery [3, 4]. In contrast, there is still a controversy about whether posterior-only surgery can achieve complete removal of the lesion and restoration of spinal stability [5, 6]. This study aims to assess the 5-year efficacy of the posterior-only debridement, internal fixation, and interbody fusion methods using titanium mesh in the surgical treatment of thoracolumbar TB with spinal epidural abscess (SEA).

## Methods

A retrospective study was done for 45 thoracolumbar TB patients with SEA. The patients were surgically treated by posterior-only debridement, internal fixation, and interbody-fusion methods using the titanium mesh. After approval from the institutional review board of Xiangya hospital of Central South University, we obtained informed consent from each participant. Data were retrieved from the hospital database for a duration from January 2008 to January 2014.

### The inclusion criteria

Diseased lesions involving (T11 – L2) region; lesions localized within two vertebral bodies; 3 or 4 vertebral bodies involved, but the number of vertebral bodies to be removed due to TB is not more than 2; intraspinal abscesses occupying the spinal canal; patients with a minimum 5-year follow-up time; and patients with complete follow-up data.

### The exclusion criteria

Patients with other spinal abnormalities such as spinal tumor; patients with incomplete follow-up data. Table 1 shows patients’ preoperative demographic data.

**Table 1:** Patients’ preoperative demographic data

Characteristic	Results
Age	37.76 ± 10.94
Sex (M/F)	27/18
VAS	7.00 ± 1.73
ESR	68.80 ± 23.63
Kyphosis deformity angle (degree)	29.36 ± 13.29
Disease location site:	
T11–12	10
T11-L1	5
T11-L2	2
T12-L1	16
T12-L2	3
L1-L2	9

*VAS* visual analog scale, *ESR* erythrocyte sedimentation rate, *M* male, *F* female, *T* thoracic vertebra, *L* lumbar vertebra

### Preoperative preparation

Several clinical tests included before surgery: X-ray, computed tomography (CT), magnetic resonance imaging (MRI), electrocardiogram, cardiopulmonary function, liver function, renal function, erythrocyte sedimentation rate (ESR), C-reactive protein, TB antibody and purified protein derivative (PPD) tests. A preoperative, two-week quadruple, anti-tuberculosis treatment regimen (isoniazid, rifampicin, ethambutol, pyrazinamide) was also given to the patients. Liver protective drugs, hypoproteinemia correction, and nutrition supplementation were also included for the patients. During preoperative anti-tuberculosis treatment, if the patient’s nerve damage had aggravated, then the surgical treatment was performed as soon as possible.

### Surgical methods

The patient was laid in the prone position on a bow-shaped shelf. After general anesthesia, the lesion segment was confirmed using a “C”-arm X-ray machine. A posterior-median surgical incision was made at the lesion segment confirmed. The diseased vertebrae and two upper and lower normal segments were exposed. The pedicle screws were placed in the two normal vertebrae adjacent to the diseased vertebrae. Decompression was performed on the side with more seriously destructed bone and larger paraspinal abscess. The titanium rod was fixed temporarily on the opposite side. The spinous process of the diseased vertebral body, the lamina of the decompressed side, and the superior and inferior articular processes were removed; the rib head and rib (approximately 5 cm) connected to the lower vertebral body were also removed. The ligamentum flavum was removed to expose and protect the dural sac and adjacent nerve roots. After performing intraspinal abscess removal and spinal cord decompression, the intervertebral space, the anterior vertebral body, and the paravertebral lesions were also removed. Next, the damaged intervertebral disc tissue, inflammatory necrosis, and dead bone were removed. Hydrogen peroxide and saline were used to clean the lesion area and the surrounding abscess. The residual bone surface corresponding to the upper and lower sides of the vertebral body cavity was flattened, and 0.3 g isoniazid and 1.0 g streptomycin were sprayed in the bone cavity and the abscess cavity. A titanium mesh of the corresponding length was cut and filled with autologous bone particles. The contralateral temporary fixation rod was properly opened, the bone graft channel was expanded, the dural sac and nerve roots were protected, and the titanium mesh was inserted through the lateral rear opening. Using a “C”-arm X-ray, it was confirmed that the titanium mesh was placed at the proper position. Next, the fixation rod was installed on both sides of the surgical site. The titanium rods on both sides were pressurized to fix the titanium mesh.

For patients with kyphosis, a two-step alternative temporary fixation was required to correct the kyphosis. Before proceeding further, we corrected the kyphosis first. Then, the titanium mesh interbody bone graft was placed according to the above steps. The lamina was covered with allogeneic bone on the lamina defect, and bone grafts were fixed. After the autologous bone and allogeneic bone were fully implanted, a drainage tube was placed, and the incision was sutured layer by layer.

### Postoperative treatment

The drainage tube was removed when the flow was less than 20 mL/24 h. Patients were provided with brace protection for 3–6 months after the operation. Anti-tuberculosis treatment was continued for 18 months. Liver protective drugs were also provided to protect the liver.

### Follow-up data and efficacy evaluation criteria

During the operation, the amount of blood loss and operation time were recorded. The discharged patients were scheduled to attend our outpatient clinic at definite intervals: every 3 months within the first year of surgery, every 6 months after the first year of surgery, and once every 2 years thereafter. We assessed back pain improvement by using a VAS score and neurological recovery by using an ASIA score. Disease healing rate was assessed by measuring the ESR level. The patients repeated radiological measurements, including X-ray, MRI, and CT. The X-ray was used to measure the kyphosis Cobb angle. During anti-tuberculosis treatment, routine blood tests, including liver and renal functions were monitored regularly.

### Statistical processing

We used SPSS 22.0 version for the statistical analysis. The preoperative, postoperative, and follow-up data of the patients were compared and were analyzed using the t-test. The rank-sum test was used when the data did not follow a normal distribution. The value of *P* < 0.05 was considered statistically significant.

## Results

**Fig. 1:**
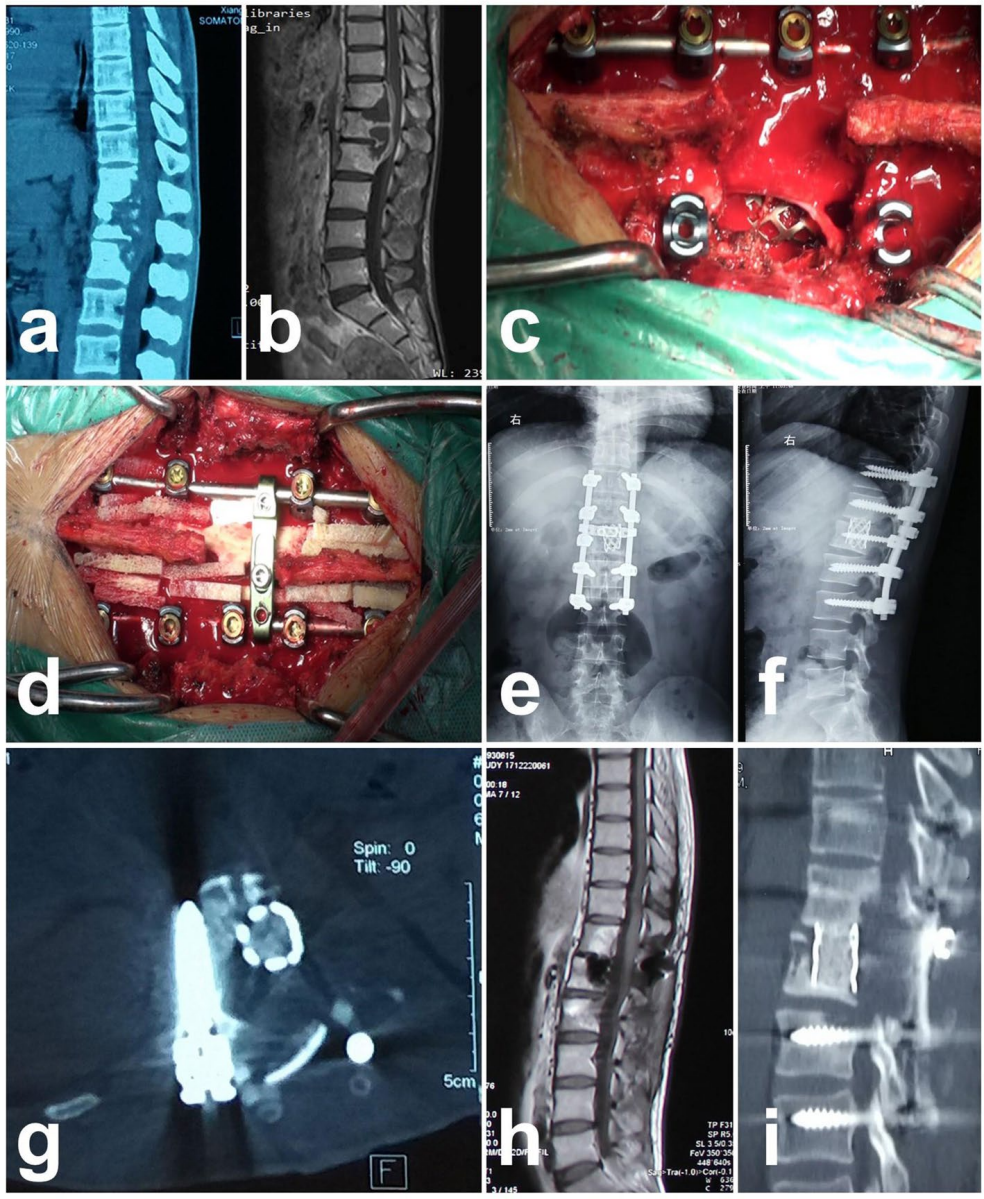
A 26-year-old male, infected with tuberculosis in the T12-L2 region. **a:** preoperative CT showed T12, L1, L2 region of the vertebral body, and intervertebral disc destruction. **b:** preoperative MRI image shows tuberculosis lesions in the spinal canal and a dural sac compression; **c:** the image shows the position of implantation of titanium mesh. **d:** the picture shows the laminar reconstruction using allogeneic bone. **e and f:** the postoperative x-ray images show good internal fixation, the implanted titanium mesh located at interbody bone defect area; **g:** postoperative CT image indicates that the titanium mesh was situated between the vertebral bodies, and the position was proper; **h:** postoperative MRI image shows a complete relief of spinal cord compression; **i:** CT sagittal view shows bone graft fusion around titanium mesh during postoperative follow-up

The operation time was 100–300 min, with an average of 199.67 ± 47.52 min. The blood loss during the operation was 180–1300 ml, with an average of 588.89 ± 263.84 ml. After surgery, 1 patient had cerebrospinal fluid leakage, 1patient had a transient neurological injury, and 1 patient had delayed healing. All these patients were cured after conservative treatment; however, one patient had tuberculosis recurrence. Therefore, we opened the wound of the patient to remove the pus and applied a drainage tube. The culture result indicated drug-resistant bacteria. The patient was cured after employing the adjusted anti-tuberculosis drug regimen. All patients had a follow-up for 60–128 months, with an average of 82.76 ± 12.56 months. The patients had a visual analog scale (VAS) score range of two weeks after surgery, with an average of 2.44 ± 0.78 points; their chest and back pain relieved in the postoperative stage than in the preoperative stage (*P* < 0.05) (Table 1). Six months after surgery, the ESR returned normal with an average of 12.13 ± 4.16 mm/h, which was significantly lower than that before surgery (*P* < 0.05). The postoperative kyphosis angle ranged − 6° – 13°, with an average of 3.58 ± 5.44°, which was lower than that before surgery (P < 0.05). Conditions at the final follow-up: the kyphosis angle was − 1° – 15°, with an average of 5.67 ± 4.80°; no significant difference between the correction degree and the postoperative correction degree (*P* > 0.05) (Table 2); no implant failures or pseudoarthrosis formations among these patients (Fig. 1); the bone graft fusion time was 6–9 months, with an average of 6.80 ± 1.34 months; postoperatively, neurological functions were restored. The ASIA classification is shown in Table 3.

**Table 2:** Perioperative and postoperative follow-up results

Variable	Results	Level of significant
Operation time (minutes)	199.67 ± 47.52	
Blood loss (ML)	588.89 ± 263.84	
Bone fusion time (months)	6.80 ± 1.34	
Follow-up time (months)	82.76 ± 12.56	
**VAS**		
Preoperative	7.00 ± 1.73	
Post-op two weeks	2.44 ± 0.78	P < 0.05, t = 14.819
**ESR**		
Preoperative	68.80 ± 23.63	
Post-op six months	12.13 ± 4.16	P < 0.05, t = 16.626
**Kyphosis (degree)**		
Preoperative	29.36 ± 13.29	
Postoperative	3.58 ± 5.44	
Final follow-up	5.67 ± 4.80	P < 0.05, t = 16.147

*ML* milliliter, *VAS* visual analogue scale, *ESR* erythrocyte sedimentation rate

**Table 3:** The value of neurological improvement according to the ASIA scale

Schedule			ASIA classification		
	A	B	C	D	E
Preoperative		3	11	16	15
Final follow-up time				7	38

*ASIA* American spinal injury association score

## Discussion

The thoracolumbar spine lacks the protection of the thorax and has a large degree of mobility due to its unique anatomical structure. The load of the spine easily induces stress concentration. As the bone is destroyed, spinal instability and deformity can easily develop in patients. An intraspinal abscess in the spinal canal will lead to spinal cord compression, which results in severe neurological deficits, numbness of the lower limbs, muscular weakness, dyskinesia, urinary or fecal incontinence, and sexual dysfunction. For these patients, even with active anti-tuberculosis drugs, surgery is often required.

The anterior approach can fully expose the lesion, so lesion removal becomes easy and anterior bone grafting also becomes convenient. However, for thoracolumbar spinal TB patients, the anterior approach requires a combined posterolateral thoracic back and abdominal incision approach. Thus, the operation procedure is very complicated and also causes massive surgical trauma. There are high risks for rupturing of pleura and injuring organs in the thoracic and abdominal cavity. The cardiac and lung functions of the patient are also impaired. Moreover, it is difficult to correct the kyphotic deformity and maintain the correct angle using a single-anterior approach [4, 7]. The single-anterior approach also leads to a risk of nerve injury; thus, making it unsuitable for long segmental fixation and spinal abscesses removal. In patients with complicated anterior fixation, it is necessary to perform posterior fixation, which further increases the surgical trauma. Single posterior surgery (SPS) can achieve lesion removal and bone graft, internal fixation, and deformity correction. Consequently, an SPS method can simplify the operation, reduce surgical trauma, and reduce the risk and cost of surgery [8, 9].

Studies have shown that for thoracolumbar spine TB patients, a single posterior approach has better clinical efficacy than the anterior or anteroposterior approach [10–12]. Though an extensive laminectomy will lead to easy decompression, lesion removal, and intervertebral bone grafting, spinal instability can occur due to extensive damage to the posterior structure. In contrast, fenestration and decompression to retain most of the posterior anatomical structure have little effect on the spine stability. However, the visual surgery field is limited and makes decompression, lesion removal, and bone grafting difficult. Patients who meet the conditions are few [13, 14].

We developed a simple posterior-only approach to remove the unilateral vertebral plate for decompression and debridement. This method can completely remove the lesion located at the vertebral body and in intervertebral space under direct vision. Anterior intervertebral bone grafting and posterior vertebral plate reconstruction increase stability of the spine [15, 16].

For thoracolumbar spinal TB with spinal canal abscess, the authors have established an operation channel by resecting the spinous process and one side of the lamina. Compared to the anterior approach, this method allows the dural sac and nerve root to be fully exposed, and the lesion to be more directly and safely removed, especially when the abscess encircles the spinal cord because it is a situation in which it would be difficult to achieve complete lesion removal through the anterior approach [17, 18]. When removing the lesion, it is necessary not only to remove the inflammatory necrosis, pus, and the diseased posterior longitudinal ligament to relieve nerve compression but also to completely remove the cheese-like substance, granulation, dead bone, and degenerative and necrotic tissue in the intervertebral space. The intervertebral area is the primary site of spinal TB. During the operation, the inflammatory tissue, dead bone, and damaged intervertebral disc of the prime central lesion were removed entirely, and the surrounding sinus was explored and irrigated. The cyst was repeatedly washed with hydrogen peroxide and saline. For satellite lesions, after the pus, caseous necrotic tissue, and tuberculous granulation tissue removal, the healthy bone surface was scraped with a curette. The primary lesions of adult spinal TB are often located in the intervertebral space, and the intervertebral space lesions must be removed entirely. This is the key to prevent recurrence after surgical treatment.

After thorough removal of the tuberculosis lesions, reliable reconstruction of the anterior vertebral body is considered essential for the success of the operation. The anterior approach is often selected because anterior bone grafting is convenient [19, 20], but a combined chest and abdominal incision is required, which causes massive trauma.

This incision is suitable for intervertebral bone grafting if performing an extensive posterior laminectomy; however, it will inevitably cause excessive destruction of the posterior column, which damages the stability of the spine. Resecting one side of the lamina mostly maintains the stability of the posterior column, while fixing the posterior pedicle screw and the titanium mesh on the anterior side of the column provide strong support to balance the spine. Therefore, surgery will have little effect on spine stability. The stability of the spine was reconstructed with bony fusion achieved due to interbody fusion and interlaminar bone fusion. We used titanium mesh for anterior intervertebral bone grafting. The autologous bone particles in the titanium mesh came from the posterior lamina, spinous processes, and ribs. When the autologous bone was insufficient, the titanium mesh was filled with allogeneic bone particles. However, we ensured that the bone grafting area was in contact with autologous bone, which is a good condition for bone fusion. After placing the titanium mesh, the intervertebral space was appropriately enlarged; hence, the titanium mesh was set and was adequately pressed against the gap to achieve immediate stability. Also, the deformity could also be corrected by avoiding movement and displacement of the titanium mesh. Titanium mesh can be selected and constructed according to the anterior defect area, and a single large titanium mesh is always implanted. However, when a single large titanium mesh is difficult to implant, multiple shaped titanium meshes can be selected to facilitate anterior interbody fusion [21, 22]. For all patients in our study, the kyphosis angle was significantly corrected, from an average of 29.36 ± 13.29° before surgery to an average of 3.58 ± 5.44° after surgery. At the last follow-up, there was no significant loss of correction angle. All patients achieved bony fusion without loosening or collapsing the titanium mesh.

The study has two limitations: a retrospective study, a single-centered study with small sample size. We call upon multicenter studies with a large sample size to be carried out in the future for further verification of our findings.

## Conclusion

For thoracolumbar spinal TB with intraspinal abscess, single posterior debridement, titanium mesh interbody fusion, and internal fixation can fully decompress the spinal cord, clear the lesion, and provide robust spinal reconstruction. This approach avoids interference with anterior organs in the abdominal cavity and chest cavity and is an effective and safe surgical procedure.

## Supplementary Information

**Additional file 1.**

## Data Availability

“All datasets analyzed during this study are included in this article given as supplementary information file 1”.

## Abbreviations

TB: Tuberculosis
ASIA: American Spinal Injury Association
SPSS: Statistical Package for the Social Sciences
M: Male
F: Female
VAS: Visual analog scale
ESR: Erythrocyte sedimentation rate
T: Thoracic vertebra
L: Lumbar vertebra
CT: Computed tomography
MRI: Magnetic resonance imaging
PPD: Purified protein derivative
ML: Milliliter
